# Status of intestinal parasitic infections among residents of Jimma Town, Ethiopia

**DOI:** 10.1186/1756-0500-7-502

**Published:** 2014-08-07

**Authors:** Ayalew Jejaw, Ahmed Zeynudin, Endalew Zemene, Tariku Belay

**Affiliations:** Department of Biomedical Science, College of Health Sciences, Mizan Tepi University, Mizan Teferi, Ethiopia; Department of Medical Laboratory Sciences and Pathology, College of Public Health and Medical Sciences, Jimma University, Jimma, Ethiopia

**Keywords:** Intestinal parasites, Prevalence, Jimma Town, Ethiopia

## Abstract

**Background:**

Intestinal parasites cause considerable morbidity and mortality in the world, especially in developing countries like Ethiopia. Both urban and rural inhabitants are vulnerable to infection with intestinal parasites in developing countries. The aim of this study was to determine the status of intestinal parasitic infections (IPIs) among residents of Jimma Town, seven years after high prevalence was reported.

**Results:**

Four hundred and thirty four residents of Jimma Town were included in this study. By the cross-sectional survey, the overall prevalence of intestinal parasites was 209 (48.2%). Nine species of intestinal parasites were isolated, *Ascaris lumbricoides* and *Trichuris trichiura* being the most predominant. Residence in Hermata Mentina *kebele*, Adjusted Odds Ratio (AOR), 3.0, 95% CI, 1.71-5.39), age less than 10 years (AOR, 3.7, 95% CI, 1.33-10.36), illiteracy (AOR, 3.2, 95% CI, 1.64-6.19), estimated monthly family income of less than 500 Ethiopian Birr (AOR, 2.9, 95% CI, 1.32-4.90) and irregular washing hands before meal (AOR, 5.3, 95% CI, 1.36-21.07) were predictors of IPI in this study. The retrospective study revealed a significant decrease (P = 0.037) in the proportion of patients infected with intestinal parasites out of those who requested stool examination over the six-year period.

**Conclusion:**

This study confirms that IPIs are still common among residents of Jimma Town. Nearly half of the study participants were infected with at least one intestinal parasite. Public health interventions targeting prevention of IPIs should be strengthened in Jimma Town.

## Background

Intestinal parasitic infections (IPIs) are among the major health problems in developing countries. Human intestinal parasites, caused by intestinal helminthic and protozoan parasites, result in significant morbidity and mortality in endemic countries. Globally, in 2010, an estimated 819.0 million people were infected with *Ascaris lumbricoides*, 438.9 million with the hookworms and 464.6 million *Trichuris trichiura* [1]. These soil transmitted helminthes (STHs) disproportionately affect people in developing countries, where sanitary facilities and clean water supply are scarce. In developed countries, the intestinal protozoan parasites are more common than the STHs [2–5].

Giardiasis, amebiasis, cryptosporidiosis, cyclosporidiosis and isosporiasis are the common parasitic protozoan diseases of significant importance. The microbial agents causing these diseases are mainly waterborne [6]. The coccidian parasites *Cryptosporidum* species, *Cyclospora cayetenensis*, *Isospora belli* and *Blastocystis hominis* are opportunistic intestinal parasites of significant importance in HIV/AIDS patients [7, 8].

Intestinal parasites affect people of all age; however, the impact on young children and pregnant women is severe. Polyparasitism and moderate-to-heavy infection with the STHs could be associated with poor growth in children [9–11] and reduced cognitive function [12], which could be reversible after deworming [13]. Association of giardiasis with vitamin A deficiency in school children has been documented [14]. In pregnant women, the STHs increase the risk of anemia [15, 16]. Hookworm infections result in significant reduction on iron store in pregnant women [17].

Ethiopia is a developing country where IPIs are major public health problems. Previous studies carried out in Ethiopia revealed high prevalence of IPIs [18, 19]. The burden of intestinal parasites, particularly the STHs, is often very high in school children [20, 21]. Amoebiasis and giardiasis are also common parasitic diseases in Ethiopia, despite the diagnostic inaccuracy in the routine laboratory diagnosis of amebiasis [22].

At the end of 2004, prevalence of intestinal parasites among residents in Jimma Town was 83% [23]. The study was a community-based cross-sectional survey, in which six of the 13 *kebeles* of Jimma Town were included. Since then, interventions by urban health extension workers (UHEWs) aimed at improving sanitary condition of the town, is being carried out. Environmental hygiene and sanitation is one of the four health subprograms of the health extension program (HEP). Thus, the health extension workers provide health information at household level to create awareness and improve skills in dealing with preventable diseases. According to the information obtained from Jimma Town Health Office, the UHEWs are involved in school water, sanitation and hygiene and awareness creation at household level to access health services, among others. As a result of introduction of the program, primary health care coverage of the country has improved [24]. The present study is aimed at determining the status of IPIs among residents of Jimma Town, seven years after 83% prevalence was documented.

## Methods

### Study area

The study was conducted in Jimma Town, located 350 kms south-west of Addis Ababa. The town is divided into 13 *kebeles* (smallest governmental administrative units in Ethiopia). The geographical coordinates of the town are approximately 7°41′ N latitude and 36° 50′ E longitude. The town is located at average altitude of 1,780 meters above sea level. According to the 2007 Central Statistical Agency census report, the projected total population of the town is 134,040. The town is generally characterized by warm climate with mean annual maximum temperature of 30°C and mean annual minimum temperature of 14°C. The annual rainfall ranges from 1138 to 1690 mm. Four public health centers, two public hospitals and several private clinics are currently found in Jimma Town.

### Study design and sampling

Community-based cross-sectional survey of intestinal parasites was conducted in selected *kebeles* of Jimma Town from January to March 2012. Moreover, retrospective study of intestinal parasites was carried out by reviewing six-year laboratory records of the public health facilities in Jimma Town. Sample size for the survey was estimated using the single population proportion formula, considering prevalence (P) of 83% from the previous study [23], 95% confidence level and 5% margin of error. This gave us sample size of 217. After multiplying it by two to account for the design effect, the final sample size was calculated to be 434 individuals. The source population for the study was residents of Jimma Town of all ages and both sexes.

A multistage sampling technique was employed. Four of the thirteen *kebeles* were selected by simple random sampling. Then, the sample size was allocated to the selected *kebeles* proportional to the total number of households in each *kebele*. Accordingly, 184, 81, 78 and 91 study participants were allocated to Bocho Bore, Bosa Addis, Seto Semero and Hermata Mentina *kebeles,* respectively. Households were selected from each of the *kebeles* by systematic sampling using list of the households as sampling frame. Finally, one individual was randomly selected from each of the selected households and included in the study.

The cross-sectional survey in our study is similar with the previous study done in Jimma [23] with respect to: i) data collection methods, ii) diagnostic methods used (direct saline smear and formol-ether concentration techniques), ii) profile of study participants included (residents of Jimma Town of all ages and both sexes). However, our study involved smaller number of study participants and *kebeles* compared to the previous study.

### Data collection and laboratory processing

Socio-demographic profile and data on risk factors associated with IPI were gathered by house-to-house interview using pre-tested questionnaire. Trained UHEWs who were conversant of the local languages (*Amharic* and *Afan Oromo*) interviewed the study participants. The questionnaire was first developed in English and then translated to *Afan Oromo* and *Amharic* by native speakers of the languages. In addition to the questionnaire data, single stool specimen was collected from each of the study participants and processed. The stool specimens were examined using direct saline smear and formol-ether concentration methods [25]. The direct saline smears were examined immediately at the nearby *kebeles* and health centers, while portion of the specimens was preserved with 10% formaline and transported to Jimma University Medical Parasitology laboratory for formol-ether concentration. Experienced laboratory technologists processed and examined the specimens.

Besides the cross-sectional survey, six-year laboratory records of intestinal parasites in the public health facilities were reviewed retrospectively. Data of patients who sought stool examination in the health care centers during the six years were extracted. The extracted data include: 1) date of stool examination; 2) total number of patients who requested stool examination 3) result of the stool examination and 4) species of intestinal parasites isolated. In Ethiopia, majority of the public health facilities do the direct wet mount as a routine laboratory diagnosis of IPIs.

### Data analysis

Data were entered, cleaned and analyzed using SPSS version 16.0 software package. Descriptive statistics were used to summarize demographic profile of the study participants. Bivariate and multivariate analyses were used to assess the association between explanatory variables and the outcome variable. The magnitude of the association was measured using adjusted odds ratio (AOR) and 95% confidence interval (CI). A P-value < 0.05 was considered as significant.

### Ethical considerations

Ethical clearance was obtained from Jimma University Research and Ethics Review Committee and permission was obtained from Jimma Zone Health Office. Informed consent was sought from each of the adult study participants and guardians/parents in case of children aged less than 18 years, prior to data collection. Information collected from the study participants was kept confidential. During the survey, individuals found to be infected with intestinal parasites were treated according to the national guideline.

## Results

### Socio-demographic characteristics of the study participants

A total of 434 study participants had participated in this study. Most of the study participants (64.1%) were female (Table 1). Age of the study participants ranged from 1 to 79 years with mean age of 25.3 years.

**Table 1:** **Characteristics of the study participants, Jimma Town, Southwest Ethiopia, 2012**

Characteristics		Frequency (%)
***Kebele***	Bocho Bore	184 (42.4)
	Hermata Mentina	91 (20.9)
	Bossa Addis	81 (18.7)
	Seto Semero	78 (18.0)
**Gender**	Male	156 (35.9)
	Female	278 (64.1)
**Occupation**	Private	44 (10.1)
	Employed	46 (10.6)
	House wife	101 (23.3)
	Daily labourer	40 (9.2)
	Student	196 (45.2)
	Others	7 (1.6)
**Age group in years**	≤10	93 (21.4)
	11-20	120 (27.6)
	21-30	88 (20.3)
	31-40	65 (15.0)
	>40	68 (15.7)
**Educational status**	Illiterate	61 (14.0)
	Literate	373 (86.0)
**Latrine availability**	Present	358 (82.3)
	Absent	76 (17.7)
**Latrine with hand washing facility (n = 358)**	Yes	60 (16.8)
	No	298 (83.2)

### Prevalence of intestinal parasites

**Figure 1:**
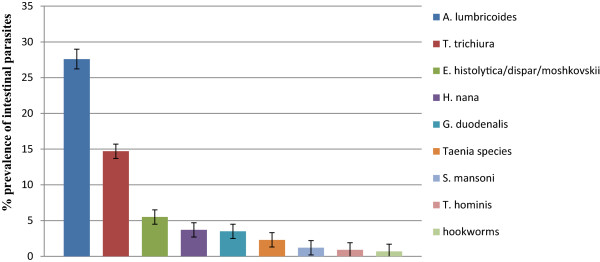
**Prevalence and types of intestinal parasites identified among residents in Jimma Town, 2012.**

Out of the 434 study participants, 209 (48.2%) were positive for at least one species of intestinal parasite. *A. lumbricoides* was the predominant parasite, detected in 120 (27.6%) of the study participants followed by *T. trichiura* (Figure 1). *Entamoeba histolytica/dispar/moshkovskii* was the predominant protozoan parasite detected in 24 (5.5%) of the study participants.

One hundred and sixty eight (80%) and 41 (20%) of the infected individuals had single and multiple parasite infections, respectively. Multiple infections were detected in 9.5% of the total study participants. The multiple intestinal parasite infections were mainly due to *A. lumbricoides, T. trichiura* and *E. histolytica/dispar/moshkovskii*.

**Figure 2:**
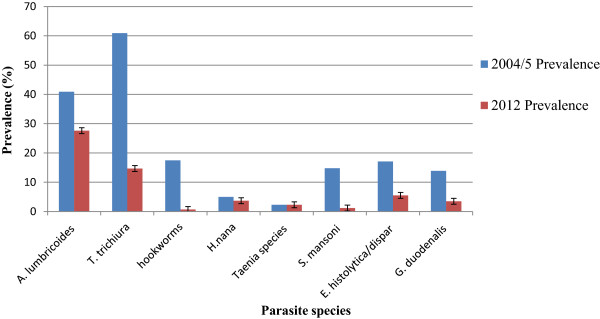
**Comparison on prevalence of each of the intestinal parasites in 2004/05 and 2012.**

Decline in prevalence of each of the intestinal parasites, except *Taenia* species which remained the same, was noted in this study compared to the previous study. The STHs *T. trichiura*, *A. lumbricoides* and hookworms were predominant in the previous study. In this study, while *A. lumbricoides* and *T. trichiura* are still predominant hookworm remarkably decreased from 17.5% in 2004/05 to 0.7% in this study (Figure 2).

Factors associated with IPIs were demonstrated in Table 2. Males were slightly more infected than females. After adjusting for possible confounding variables (Table 2), residence in Hermata Mentina *kebele* (AOR = 3.037, 95% CI: 1.71-5.39), age group less than 10 years (AOR = 3.718, 95% CI: 1.33-10.36), illiteracy (AOR = 3.196, 95% CI: 1.64-6.19), estimated family income of less than 500 Ethiopian birr (AOR = 2.875, 95% CI: 1.32-4.90) and irregular washing of hands before meal (AOR = 5.355, 95% CI: 1.36-21.07) were significantly associated with IPIs.

**Table 2:** **Factors associated with intestinal parasite infection among residents in Jimma Town, Southwest Ethiopia, 2012**

Characteristics		Intestinal parasite		COR (95%CI)	AOR (95%CI)	
		Positive n(%)	Negative n(%)			P-value
***Kebele***	Bocho Bore	87(47.3)	97(52.7)	1	1	<0.001
	Hermata Mentina	59(64.8)	32(35.2)	2.1(1.22-3.45)*	3.0(1.71-5.39)	
	Seto Semero	32(41)	46(59)	0.8(0.45-1.32)	0.8(0.42-1.40)	
	Bossa Addis	31(38.3)	50(61.7)	0.7(0.40-1.17)	0.7(0.40-1.33)	
**Sex**	Male	83(53.2)	73(46.8)	1.4(0.92-2.03)	1.5 (0.931-2.27)	>0.05
	Female	126(45.3)	152(54.7)	1	1	
**Age group in years**	<10	60(64.5)	33(35.5)	1.6(0.85-2.82)	3.8(1.33-10.36)	<0.001
	10-20	64(53.3)	56(46.7)	1	1	
	21-30	40(45.5)	48(54.5)	0.7(0.42-1.26)	0.8(0.45-1.53)	
	31-40	25(38.5)	40(61.5)	0.6(0.29-1.01)	0.6(0.28-1.10)	
	>40	20(29.4)	48(70.6)	0.4(0.19-.68)*	0.3(0.13-.55)	
**Educational status**	Illiterate	37(60.7)	24(39.3)	1.8(1.03-3.13)*	3.2(1.64-6.19)	<0.001
	Literate	172(46.1)	201(53.9)	1	1	
Monthly family income**	<500	155(52.9)	138(47.1)	1.5(0.76-2.764)*	2.9(1.32-4.90)	0.041
	≥500	39(37.9)	64(62.1)	1	1	
**Hand washing before meals**	Always	196(46.9)	222(53.1)	1	1	0.016
	Sometimes	13(81.3)	3(18.7)	4.9(1.37-17.47)*	5.4(1.36-21.07)	
**Solid waste disposal method**	Burning	128(44.9)	157(55.1)	1	1	>0.05
	Pit	67(54.9)	55(45.1)	1.5(0.976-2.288)	1.3(0.809-2.114)	
	Open field	14(51.9)	13(48.1)	1.3(0.599-2.911)	1.4(0.568-3.293)	

*Statistically significant at P < 0.05, COR = Crude Odds Ratio, AOR = Adjusted Odds Ratio, adjusted for other factors shown in the table, CI = Confidence Interval **Estimated monthly family income in Ethiopian Birr.

### Retrospective record review

**Figure 3:**
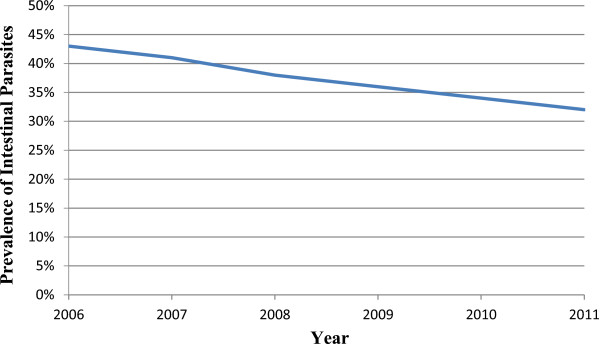
**Annual trend of prevalence of intestinal parasites documented in the public health facilities in Jimma Town, 2012.**

Laboratory records of patients who requested stool examination in the public health institutions during the six years were reviewed retrospectively. Accordingly, the proportion of patients positive for intestinal parasites decreased from 43.34% in 2006 to 31.83% in 2011. During these periods, a total of 69,782 stool specimens had been examined in the health facilities. Overall, 36.22% of the stool specimens were positive for at least one species of intestinal parasite. The reduction in the proportion of patients positive for intestinal parasites was significant (P = 0.037) (Figure 3).

## Discussion

The overall prevalence of intestinal parasites among residents in Jimma Town in this study was 48.2%. The overall prevalence of intestinal parasites declined from 83% at the end of 2004 [23] to 48.2% in this study. The marked reduction in the overall prevalence of intestinal parasites could be the outcome of introduction of the HEP. Ethiopian Ministry of Health launched Rural Health Extension Program in 2003 and the Urban Health Extension Program in 2009 [26]. The program has a defined package of basic and essential promotive, preventive and selected high impact curative health services targeting households.

Despite the remarkable reduction in the overall prevalence of intestinal parasites compared to the previous study, nearly half (48.2%) of the residents were infected by at least one species of intestinal parasite. This shows that intestinal parasites still pose significant burden to residents of Jimma Town. The finding of this study calls for urgent interventions particularly targeting school-age children, as the prevalence of intestinal parasites is disproportionately higher in this age group. Preventive chemotherapy to school-age children is required as the STHs could result in significant nutritional impact [27] and poor cognitive performance [28].

Our study investigated several possible factors associated with IPIs and identified predicators of infection. Accordingly, illiteracy, low family income and irregular washing of hands before meal were predicators of IPIs in this study. This shows the role of education in preventing IPIs and keeping personal hygiene practices. The significant association of low family income with IPIs may be related to lack of pure water supply and sanitation facilities. Several studies also documented association of IPIs to poor personal hygiene, lack of sanitary facilities and poor economic status [29, 30]. The prevalence of *Taenia* species remained the same (2.3%) in the previous and this study. The dietary habit of consuming raw meat as in *kitfo* and *kurt* in Ethiopia in general and Jimma Town in particular likely contributed for this.

The following limitations of our study should be considered when interpreting the results. Intensity of the STHs has not been determined. As a result, severity of STH infections could not be shown. Moreover, the prevalence data generated by the retrospective study represents data of patients who sought stool examination at the health facilities, thus, it could not be generalized to the general population.

## Conclusion

There was a significant decrease in overall prevalence of intestinal parasites over the six-year period. However, nearly half of the study participants harbored at least one species of intestinal parasite, *A. lumbricoides* and *T. trichiura* being the most predominant. Control efforts should focus on provision of health information to improve personal hygiene practices and properly dispose human excreta. School-based awareness creation on the modes of transmission of intestinal parasites is recommended.
